# Novel Egg White Protein–Chitin Nanocrystal Biocomposite Films with Enhanced Functional Properties

**DOI:** 10.3390/polym17182538

**Published:** 2025-09-19

**Authors:** Víctor Baquero-Aznar, Víctor Calvo, José Miguel González-Domínguez, Wolfgang K. Maser, Ana M. Benito, María Luisa Salvador, Jaime González-Buesa

**Affiliations:** 1 Departamento de Ciencia Vegetal, Instituto Agroalimentario de Aragón—IA2, Centro de Investigación y Tecnología Agroalimentaria de Aragón (CITA), Av. Montañana 930, 50059 Zaragoza, Spain; vbaquero@cita-aragon.es (V.B.-A.); jgonzalez@cita-aragon.es (J.G.-B.); 2 Grupo de Investigación en Alimentos de Origen Vegetal, Instituto Agroalimentario de Aragón—IA2, Universidad de Zaragoza, Miguel Servet 177, 50013 Zaragoza, Spain; 3 Instituto de Carboquímica (ICB), Consejo Superior de Investigaciones Científicas (CSIC), Miguel Luesma Castán 4, 50018 Zaragoza, Spain; vcalvo@icb.csic.es (V.C.); jmgonzalez@icb.csic.es (J.M.G.-D.); wmaser@icb.csic.es (W.K.M.); abenito@icb.csic.es (A.M.B.)

**Keywords:** chitin nanocrystals, egg white protein films, compression molding, bio-nanocomposites, mechanical properties

## Abstract

This study aims to develop egg white protein (EWP) biocomposite films reinforced with chitin nanocrystals (ChNCs, 1–5 wt.%) by compression molding to overcome the mechanical and barrier limitations of protein-based films for sustainable packaging. ChNC incorporation may modulate film microstructure, crystallinity, and thermal stability, thereby enhancing functional performance. Films were prepared by adding ChNCs either as aqueous suspensions or lyophilized powder, and their structural, thermal, mechanical, optical, and barrier properties were systematically evaluated. Scanning electron microscopy confirmed a more homogeneous dispersion of ChNCs when added as suspensions, while powder addition promoted partial aggregation. X-ray diffraction revealed increased crystallinity with ChNC reinforcement. Mechanical tests showed that films with 2 wt.% ChNCs in suspension exhibited the highest tensile strength, whereas those with 5 wt.% in powder form became stiffer but less extensible. Oxygen permeability was not significantly affected, while water vapor permeability decreased by up to 14.5% at 2 wt.% ChNCs incorporated as powder. Transparency and color remained largely unchanged by ChNC addition, except for a slight increase in yellowness. Overall, these findings demonstrate that the incorporation method and concentration of ChNCs play a crucial role in tailoring the physicochemical performance of EWP films. The results provide new insights into the design of EWP-based nanocomposites and support their potential as bio-derived materials for advanced food packaging applications.

## 1. Introduction

Rising worldwide concern about plastic pollution and the ecological footprint of petroleum-derived packaging has stimulated extensive efforts toward identifying sustainable alternatives. Among these, natural biopolymers, particularly polysaccharides and proteins, have attracted significant interest because of their biodegradability, renewability, biocompatibility, non-toxicity, and their potential to substitute conventional plastics across diverse applications, most notably in food packaging [1,2,3,4,5,6].

Protein-based materials represent a particularly interesting class of biopolymers by virtue of their good film-forming performance [7,8,9,10], oxygen barrier capabilities [9,11], and ability to incorporate functional additives [12,13,14,15,16,17]. However, their performance is often hindered by limited moisture barrier performance, attributable to the hydrophilic character of the amino acids in its structure [18,19,20,21,22], mechanical brittleness [10,23], and sensitivity to moisture and temperature [4,24]. These drawbacks significantly constrain their broader industrial application. Several approaches have been explored in order to overcome these limitations including: blending protein-based polymers with other biopolymers for improving their physicochemical properties and functional performance [25,26], multilayer films in which each layer plays a specific role [27,28], the use of plasticizers [29,30], enzymatic [31,32,33], chemical and physical crosslinking [23,34,35,36,37,38], and the incorporation of nanomaterials and functionalized nanomaterials to form nanocomposite structures [10,17,39,40,41,42,43].

Integrating nanoscale reinforcements within the polymer matrix enables synergistic interactions with polymer chains, which significantly enhance the material’s mechanical performance, including stiffness, tensile strength, and flexibility [44,45,46]. Additionally, nanofillers may reduce permeability [47,48,49], improve thermal stability [50] and functional properties [13,17,41,43] of the resulting films. A wide range of nanomaterials have been studied for this purpose, including nanoclays (e.g., montmorillonite), metal and metal oxide nanoparticles, cellulose nanocrystals, and chitin nanocrystals (ChNCs) among others.

ChNCs, in particular, offer several advantages that make them highly suitable for inclusion in nanobiocomposite films [48]. Commonly derived from the acid hydrolysis of chitin—the second most common polysaccharide in nature, with crustacean shells being its primary source —ChNCs combine biocompatibility, high aspect ratio, and crystallinity, which contribute to excellent mechanical reinforcement and structural stability [51,52]. The enhancement of mechanical properties in food biopolymer films through the incorporation of ChNCs is primarily attributed to its high specific surface area and strong chemical compatibility with polymeric matrices. Moreover, the presence of ChNCs contributes to increased structural tortuosity within the film network, which effectively reduces the diffusion of gas molecules and, consequently, improves barrier performance [39,48]. In addition, they offer unique functionalities conferred by their natural antimicrobial activity, ascribed to the acetamido groups present on their surface [53].

The potential of protein-based nanocomposites containing ChNCs has been primarily evaluated in gelatin-based films. Incorporation of ChNCs had been shown to improve thermal stability and tensile strength [12,24,54,55,56], reduce solubility and water vapor permeability [55,56], and increase surface hydrophobicity [56]. Nevertheless, in certain cases, a decrease in flexibility and transparency was observed in the resulting films [24]. Furthermore, the incorporation of ChNCs has been reported to improve the antimicrobial properties of gelatin films, either on their own [56,57] or through a synergistic effect when combined with ZnO nanoparticles [54]. Comparable results were observed in zein-based films, where the addition of ChNCs led to improved tensile strength and antimicrobial functionality, particularly when combined with cinnamon essential oil [58]. In addition, zein/potato starch films containing chitosan/curcumin nanoparticles showed improved mechanical strength and water vapor barrier capacity with increasing nanoparticle content, leading to films that possessed both antimicrobial and antioxidant functions [59].

The number of studies involving the development of egg white protein (EWP)-based nanocomposite films are limited, basically because the development of EWP films has lagged behind that of other protein-based systems [60]. Nevertheless, egg white protein (EWP) is an easily accessible protein source with high nutritional value and excellent functional and processing properties [61,62,63]. These characteristics render EWP a promising candidate for the development of food packaging films [64]. EWP-based films exhibit mechanical and barrier properties comparable to those of films derived from other proteins, while offering superior transparency [64,65,66,67]. The development of clay-based EWP nanocomposites has also showed promising results. Diañez et al. [65,68] demonstrated that obtaining a uniform distribution of nanoclay throughout the EWP network leads to films with increased stiffness, higher resistance to deformation, and superior gas barrier performance against oxygen and carbon dioxide. Similarly, Giménez et al. [69] reported enhancements in Young’s modulus and tensile strength when sepiolite was incorporated into gelatin–EWP composite films. However, as far as we are aware, the influence of ChNCs on the properties of EWP-based films has not been explored to date.

The aim of this study was to investigate the reinforcing effect of ChNCs on EWP-based nanobiocomposite films and to evaluate their potential for food packaging applications. In this context, the novelty of the present work lies in evaluating, for the first time, the incorporation of ChNCs into EWP-based films assessing their influence on functional properties, which distinguishes this study from previously reported biopolymer composites. The working hypothesis was that the addition of ChNCs to EWP films may favorably modify the film microstructure, crystallinity, and thermal stability, thereby improving their functional properties, specifically mechanical and barrier properties. To this end, films were prepared with different ChNC concentrations, incorporated into the film-forming solutions either as aqueous suspensions or as lyophilized powder. The structure and morphology of the compression-molded films were examined through scanning electron microscopy (SEM), X-ray diffraction (XRD), and thermogravimetric analysis (TGA). Furthermore, their optical and mechanical properties, together with their oxygen and water vapor permeability, were systematically evaluated.

## 2. Materials and Methods

### 2.1. Materials

Egg white protein (EWP) powder was kindly supplied by Bouwhuis Enthoven BV (Raalte, The Netherlands). Food-grade glycerin (GLY) was obtained from Barcelonesa Global Chemical Solutions (Cornellà de Llobregat, Spain). Practical grade chitin powder from shrimp shells was obtained from Sigma-Aldrich (St. Louis, MO, USA). Analytical grade hydrochloric acid (37%) was obtained from Labbox Labware (Premià de Dalt, Spain).

### 2.2. Chitin Nanocrystals (ChNCs) Synthesis

ChNCs were prepared through hydrochloric acid hydrolysis, following the method reported by Calvo et al. [70], derived from the protocol of Narkevicius et al. [71]. Chitin (4 g) was hydrolyzed in 80 mL of 3 M HCl under reflux for 90 min using a heating plate set to 120 °C, after which the reaction was terminated by transferring the mixture into 1 L of ice-cold ultrapure water. After overnight storage at 4 °C, the sediment was separated by decantation and dialyzed against ultrapure water using a 33 mm flat width regenerated cellulose dialysis membrane (D9652, Merck KGaA, Darmstadt, Germany) until neutral pH was achieved. In sequence, the suspension was sonicated with a 24 kHz DRH-P400S ultrasonic processor (Hielscher Ultrasonics GmbH, Teltow, Germany) set to 20% amplitude and 50% cycle time for 20 min. The sample was then centrifuged at 9000 rpm for 20 min, and the supernatant was collected. The remaining pellet was redispersed in ultrapure water (40 mL per tube, using six Falcon tubes) and centrifuged again under identical conditions. This redispersion-centrifugation cycle was repeated twice to ensure complete recovery of ChNCs. These dispersions were stored either as a liquid at 4 °C or as a solid powder after lyophilization. The resulting products were characterized by FTIR, XRD and TGA.

### 2.3. EWP Films Preparation

A mixture of EWP, glycerol, and water in a 2:1:1 *w*/*w* ratio was prepared to obtain the film forming solutions (FFS). The protein-to-(glycerol plus water) ratio was selected based on the gelling capacity of egg white protein (EWP), as determined by the results of previous studies [64]. The loading level of ChNCs was calculated relative to the EWP weight and incorporated into the mixture either as a water-dispersed solution (1% and 2% *w*/*w*) or as a lyophilized powder (2% and 5% *w*/*w*). All ingredients were mixed in a mortar and pestle for 10 min under ambient conditions, and the FFSs obtained were loaded in 20 mL syringes and stabilized at 4 °C for at least 24 h. After that, 10 g of the FFSs were distributed between two aluminum foils and were introduced in a LP-S-50 laboratory hot press (Labtech Engineering Company Ltd., Phraeksa, Thailand). Compression molding was performed in two cycles: an initial heating cycle at 130 °C and 150 bar for 5 min, followed by a cooling cycle at 20 °C for 5 min, while maintaining the same pressure. The resulting EWP films were gently removed from the aluminum foils and conditioned at 23 °C and 55% RH for 48 h prior to use. For each condition, three FFS replicates were prepared, producing two films from each replicate.

### 2.4. Rheological Behavior of Film-Forming Solution

Rheological measurements of FFS samples equilibrated to room temperature were carried out with an MCR301 rheometer (Anton Paar Physica, Graz, Austria) using a serrated parallel plate geometry (50 mm diameter, 1 mm gap). Rotational tests were conducted to assess the effect of temperature on FFS viscosity. Each sample was first conditioned at 25 °C for 5 min under a constant shear rate of 10 s^−1^. Thereafter, the temperature was raised at 5 °C·min^−1^ to 130 °C, while keeping a constant shear rate of 50 s^−1^ (a value previously determined to ensure operation within the linear viscoelastic region, based on shear rate sweep experiments). The viscoelastic response of FFS as a function of temperature was further examined through oscillatory tests conducted at a constant frequency of 0.5 Hz and a strain of 1%, selected based on previous studies. The oscillatory temperature ramp began with a 5 min stabilization period at 25 °C, followed by a continuous heating phase from 25 °C to 105 °C, at a rate of 5 °C·min^−1^. All rheological assessments were conducted in triplicate using three independent FFS preparations.

### 2.5. EWP Films Characterization

#### 2.5.1. Fourier Transform Infrared Spectroscopy (FTIR)

The FTIR analysis of EWP films, both neat and reinforced with ChNCs, was performed on a Bruker Vertex 70 spectrometer (Bruker Corporation, Billerica, MA, USA) fitted with an attenuated total reflectance accessory. Spectra were recorded in the 4000–550 cm^−1^ region, with 32 scans averaged per sample at a resolution of 4 cm^−1^.

#### 2.5.2. X-Ray Diffraction (XRD)

Neat and ChNCs-reinforced EWP films were examined by XRD with a Bruker D8 Advance diffractometer (Bruker Corporation, Billerica, MA, USA) using Cu Kα radiation (λ = 1.54 Å), operating at 40 kV and 40 mA. Measurements were performed in Bragg−Brentano configuration across a 2θ range of 5–40°, with data collected at 0.05° step intervals and an acquisition time of 3 s per step.

#### 2.5.3. Thermal Gravimetric Analysis (TGA)

TGA measurements of the EWP films were conducted on a TG 209F1 Libra thermobalance (Netzsch, Selb, Germany) under a nitrogen flow of 60 mL min^−1^. The temperature was increased from room temperature to 800 °C at a constant heating rate of 10 °C·min^−1^.

#### 2.5.4. Scanning Electron Microscope (SEM)

The morphology of the films, both at the surface and in cross-section, was examined using field-emission scanning electron microscopy (FE-SEM). Film fragments (~0.5 cm × 0.5 cm) were fixed onto aluminum stubs with carbon adhesive tape. Cross-sections were prepared by freeze-fracturing in liquid nitrogen and similarly mounted. All samples were sputter-coated with a 14 nm palladium layer prior to imaging. SEM images were acquired through an Inspect F50 FE-SEM (Thermo Fisher Scientific, Waltham, MA, USA) set to an accelerating voltage of 10 kV.

#### 2.5.5. Transmission Electron Microscopy (TEM)

EWP films were embedded in EMBed 812 epoxy resin (Electron Microscopy Sciences, Hatfield, PA, USA) and sectioned using an EM UC7 ultramicrotome (Leica Microsystems, Vienna, Austria) fitted with a diamond blade. The resulting ultrathin slides were mounted on carbon-coated 200 mesh copper grids (Agar Scientific Ltd., Rotherham, UK) and examined using a Tecnai T20 transmission electron microscope (Thermo Fisher Scientific, Waltham, MA, USA) at an accelerating voltage of 200 kV.

#### 2.5.6. Mechanical Properties

For the evaluation of the mechanical behavior of the EWP films, tensile strength (TS), elongation at break (EAB), and Young’s modulus (E) were determined with a TA-XT2i texture analyzer (Stable Micro Systems Ltd., Godalming, UK) fitted with A/TGR tensile grips, following the ASTM D882-18 procedure [72]. TS (MPa) was calculated as the maximum force at rupture (N) divided by the initial cross-sectional area of the film (mm^2^), EAB (%) was defined as the percentage increase in length at failure relative to the original specimen length, and E (MPa) was obtained as the ratio between stress and strain at the point of rupture. Prior to testing, the film samples (10 mm wide × 90 mm long) were conditioned for at least 48 h at 23 °C and 50% RH. Tensile tests were conducted with a gauge length of 50 mm and a crosshead speed of 1 mm·s^−1^. For each EWP film formulation, a total of 15 specimens from 3 independently prepared batches were analyzed.

#### 2.5.7. Optical Properties

The color of both neat and ChNC-reinforced EWP films was evaluated with a CR-200 colorimeter (Konica Minolta Sensing Inc., Tokyo, Japan). Color measurements were expressed in the CIELab system: L* denotes lightness, and a* and b* represent the chromaticity coordinates. The films were mounted over the instrument’s standard white calibration plate (L* = 97.34; a* = 0.06; b* = 1.82) during measurement.

Film optical transmittance was measured with a Libra S22 spectrophotometer (Biochrom, Cambridge, UK) at 600 nm. Film specimens (25 mm ×10 mm) were placed flat against one face of the cuvette, with an empty cuvette as reference. For each processing condition, two measurements were performed on each of three independently prepared film replicates.

#### 2.5.8. Oxygen Permeability (OP) and Water Vapor Permeability (WVP)

The transmission rate of oxygen (OTR) and water vapor (WVTR) were determined under controlled conditions of 23 °C and 50% RH. OTR was measured using a MOCON Ox-Tran^®^ Model 2/22 (Minneapolis, MN, USA) in compliance with ASTM D3985-17 [73], whereas WVTR was evaluated with a MOCON Permatran-W^®^ Model 3/34 (Minneapolis, MN, USA) following ASTM F1249-20 [74]. At least 6 replicates were tested for both neat and ChNCs-reinforced EWP films. The permeabilities of oxygen and water vapor (*P*, kg m m^−2^ s^−1^ Pa^−1^) were obtained from their respective transmission rates (*TR*, kg m^−2^ s^−1^) by applying the following equation:

(1) $$P=\frac{TR\;\cdot e\;}{∆P}$$

where *e* is the mean film thickness (m), measured in 5 points using a digital thickness gauge (model 547-401, Mitutoyo, Japan), and ∆*P* (Pa) is the partial pressure difference of oxygen or water vapor across the film.

### 2.6. Statistical Analysis

For each factor (ChNC concentration), 6 films were prepared from 3 independently prepared FFS batches. Statistical analyses were carried out with GraphPad Prism version 10 (GraphPad Software, San Diego, CA, USA). Results are reported as mean ± standard deviation (SD). Differences between groups were evaluated by one-way ANOVA followed by Tukey’s post hoc test, considering *p* < 0.05 as the threshold for statistical significance.

## 3. Results and Discussion

### 3.1. Morphological Properties of EWP Films

**Figure 1:**
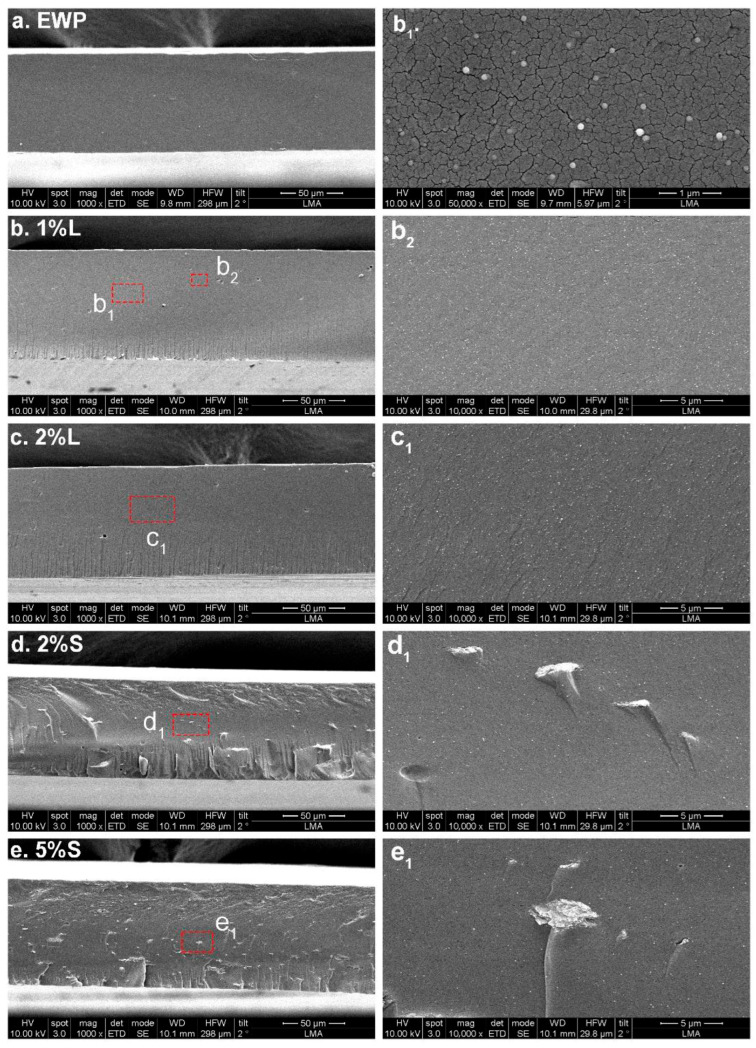
Scanning electron microscope (SEM) pictures of the cross-section microstructure of egg white protein films: (**a**) neat EWP film, (**b**) EWP film with 1% L ChNCs, (**b_1_**,**b_2_**) detailed views of (**b**) regions, (**c**) EWP film with 2% L ChNCs, (**c_1_**) detailed view of (**c**) region, (**d**) EWP film with 2% S ChNCs, (**d_1_**) detailed view of (**d**) region, (**e**) EWP film with 5% S ChNCs, (**e_1_**) detailed view of (**e**) region.

The films’ surface and cross-sectional morphological structure was analyzed by SEM. The neat EWP films displayed a uniform and smooth surface morphology, free of apparent defects (Figure S1). The incorporation of ChNCs did not produce significant changes in surface morphology (Figure S1); however, noticeable differences were observed in the cross-sectional microstructure (Figure 1). Upon the addition of ChNCs, rounded nanostructures with a diameter of ~65 nm were detected (Figure 1b_1_), consistent with the morphology of chitin nanocrystals previously reported in nanocomposite films [56,57,75,76]. When ChNCs were incorporated into FFS as an aqueous suspension, they were well dispersed and homogeneously distributed throughout the film. In contrast, the use of ChNCs in powder form resulted in less efficient dispersion, leading to the formation of larger aggregates; most pronounced in samples containing 5 wt.% ChNCs (Figure 1e). Similar aggregation behavior has been reported in starch-based nanocomposites, where low ChNCs concentrations (e.g., 2 wt.%) favored uniform dispersion, while higher contents led to nanoparticle agglomeration [77]. The aggregates observed in the SEM micrographs of samples containing powdered ChNCs were also confirmed by transmission electron microscopy (TEM), exhibiting comparable morphological features (Figure S2).

### 3.2. FTIR

**Figure 2:**
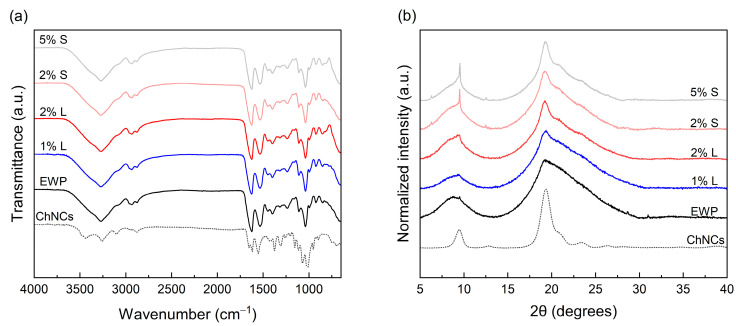
FTIR (**a**) and XRD (**b**) of egg white protein films with ChNCs in liquid (L) and solid (S) states at different concentrations.

The FTIR spectrum of ChNCs (Figure 2a) exhibits characteristic bands in agreement with previously reported values [70,78]. The broad absorption peaks observed at approximately 3440 and 3260 cm^−1^ correspond to O–H and N–H stretching vibrations, respectively [55,79,80]. The amide I bands appear at 1653 and 1620 cm^−1^, amide II at 1558 cm^−1^, and amide III at 1310 cm^−1^, in agreement with previous findings [51]. The FTIR spectrum of EWP films is also shown in Figure 2a. It displays characteristic transmittance peaks associated with protein amides: amide A (3272 cm^−1^), amide B (2961 cm^−1^), amide I (1625 cm^−1^), amide II (1533 cm^−1^), and amide III (1392 cm^−1^), consistent with earlier reports on neat EWP films [61,64].

The incorporation of ChNCs into the EWP matrix did not lead to noticeable alterations in the spectra. This has also been reported for gelatin-based films and is attributed to the low ChNC content, with weak vibrational signals being masked by the strong protein absorption bands, particularly in the amide I and II regions [24]. Similar findings have been reported for PLA [81] and carrageenan [82] nanocomposites, where spectra of reinforced films closely resemble those of their neat counterparts. However, in some systems, such as gelatin-based films, interactions between gelatin molecules and chitin nanoparticles through hydrogen bonding (involving N–H and O–H groups) have led to detectable variations in peak intensity. Furthermore, increased intensity of peaks attributed to the N–H bonds of the N-acetyl group of chitin has been observed with increasing chitin nanoparticle content [56]. In starch-based films [83,84], characteristic bands were also visible after ChNC incorporation, likely due to the absence of interfering amide I and II bands in the starch matrix. In chitosan-based nanocomposites, ChNC addition has been associated with a shift in –O–H and –N–H stretching bands to lower wavenumbers, suggesting the presence of hydrogen bonding and electrostatic interactions between chitosan and ChNCs [85]. Additional absorption bands have also been reported, particularly at high ChNC concentrations (above 20% v/v), providing evidence of their incorporation into the chitosan matrix [79].

### 3.3. XRD

Figure 2b displays the XRD patterns of ChNCs, neat EWP films, and EWP films reinforced with ChNCs. The diffractogram of ChNCs exhibits characteristic peaks at 2θ = 9.3°, 12.7°, 19.2°, 23.3° and 26.3°, corresponding to the (020), (021), (110), (130), and (013) crystalline planes, respectively. These diffraction features are consistent with those reported for crystalline α-chitin in the literature [70,71,79,86]. Neat EWP films display a broad, low-intensity diffraction profile characteristic of an amorphous arrangement. Specifically, two broad reflections at approximately 2θ = 8.5° and 19° have previously been attributed to the α-helix and β-sheet conformations of the protein secondary structure, respectively [61,64]. Upon incorporation of ChNCs into the EWP matrix, notable changes were observed in the XRD pattern. The intensity of the peaks at 2θ = 9.3° and 19.3° increased with ChNC content, and the peaks became sharper. These modifications reflect the contribution of the intrinsic crystalline domains of ChNCs to the overall structure of the composite films. The presence of these peaks aligns with those seen in the pure ChNC pattern, indicating a partial preservation of ChNC crystalline order within the protein matrix. Comparable increases in crystallinity following ChNC incorporation have also been documented in diverse bio-based films, such as protein-based [24,56,57], chitosan-based [87,88], and polysaccharide-based matrices [82,84,89].

### 3.4. TGA

**Figure 3:**
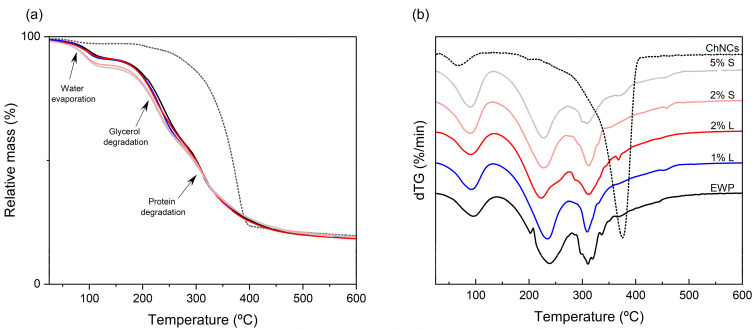
TGA (**a**) and dTG (**b**) of egg white protein films with ChNCs incorporated in liquid (L) and solid (S) states at different concentrations. Colors and line patterns indicate: ChNCs (**---**), EWP (**—**), 1% L (**—**), 2% L (**—**), 2% S (**—**), and 5% S (**—**).

Thermogravimetric (TGA) curves and their corresponding differential (dTG) analyses for ChNCs, as well as for neat and ChNCs-reinforced EWP films, are shown in Figure 3a and Figure 3b, respectively. ChNCs underwent a single degradation step, showing a peak weight-loss rate at approximately 390 °C, corresponding to the thermal decomposition of chitin [70]. All EWP films displayed three main stages of thermal degradation: (i) an initial step below 130 °C related to the release of free and bound water; (ii) a second step occurring between ~180–280 °C, ascribed to the degradation of glycerol, low-molecular-weight protein fractions, and residual bound water; and (iii) a final stage up to ~450 °C, corresponding to the breakdown of high-molecular-weight protein fractions. The degradation processes occurring during the second and third steps give rise to two dTG peaks between 223 and 238 °C and 309–312 °C. These transitions are consistent with those previously reported for protein-based films [24,56,61,90]. Although the addition of ChNCs increased crystallinity, no improvement in thermal stability was observed, likely due to nanoparticle aggregation creating microdefects that act as degradation sites, or to functional groups in the ChNCs catalyzing the process. The addition of ChNCs to the EWP matrix reduced the maximum degradation temperature of up to 15.4 °C in the intermediate region (~180–280 °C) and 2.7 °C in the final degradation stage, respectively. This effect, however, depended on the ChNC concentration and dispersion method: at 1 wt.% the shift was negligible, while at 2 wt.% films containing ChNCs dispersed in aqueous suspension exhibited less pronounced thermal changes compared to those with powdered ChNCs, likely due to a more homogeneous distribution of nanocrystals in the film-forming solution, as confirmed by SEM. The limited effect of ChNCs on thermal stability has also been noted in other protein-derived films. For instance, Etxabide et al. [24] reported minimal changes in TGA thermograms of gelatin-based films at 1–2 wt.% ChNCs, with only a 5 °C increase at 4 wt.%. Similarly, Sahraee et al. [56] observed an increase in the temperature for 4% weight loss with 5–10 wt.% ChNCs, but noted that the maximum degradation temperature actually decreased, along with an increase in residual mass. Reinforcement with ChNCs has also been shown to reduce the thermal stability of some starch-based films, attributable to the restriction in amylopectin chain flexibility imposed by the nanocrystals [83,84,89]. Conversely, in chitosan-based films, the incorporation of ChNCs has been shown to delay thermal degradation by up to 8 °C compared to neat films.

### 3.5. Rheological Behavior of FFS

**Figure 4:**
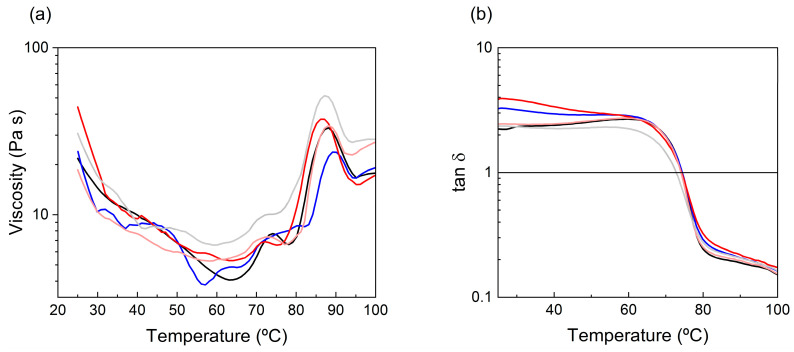
Influence of temperature on viscosity (**a**) and tan δ (**b**) in egg white protein film-forming solutions containing ChNCs, in both liquid (L) and solid (S) states at different concentrations. Colors indicate: control (**—**), 1% L (**—**), 2% L (**—**), 2% S (**—**), and 5% S (**—**).

All FFSs exhibited a typical shear-thinning behavior, consistent with previous findings for EWP-based FFSs without ChNCs [64]. As shown in Figure 4a, the viscosity decreased with rising temperature until reaching approximately 65 °C. Beyond this temperature, viscosity increased significantly, a phenomenon ascribed to thermal gelation triggered by protein denaturation, molecular unfolding, and subsequent aggregation mediated by hydrophobic forces and sulfhydryl–disulfide exchange [91,92]. These aggregation processes promote the formation of a network that restricts molecular mobility, thereby increasing viscosity [93]. Ovotransferrin, one of the thermolabile fractions in egg white, is known to denature around this temperature threshold, and is therefore likely to play a significant role in the initial increase in viscosity. A second, more pronounced rise in viscosity was detected at temperatures exceeding 85 °C, corresponding to the denaturation of ovalbumin, the most abundant egg white protein, which denatures at ~84.5 °C [94,95]. The addition of ChNCs at concentrations of 1% and 2% did not result in significant changes in the viscosity profile of the FFSs. However, at 5% ChNCs, the viscosity–temperature curve shifted upward, indicating increased resistance to flow throughout the thermal ramp. This behavior is likely due to enhanced hydrogen bonding between ChNCs and protein chains, which increases viscosity by restricting polymer chain mobility [56]. This type of ChNC-induced viscosity enhancement has also been reported in polymeric nanocomposites such as PHB [96,97] and PLA matrices [80]. Moreover, shear viscosity has been described as strongly dependent on ChNC content, increasing up to a critical threshold (typically between 10 and 15%), beyond which viscosity may decrease due to nanofiller saturation or aggregation [53].

Oscillatory rheological tests (Figure 4b) revealed a viscoelastic transition with increasing temperature. Initially, the FFS exhibited a mainly viscous behavior with tan δ > 1 (G″ > G′), transitioning to an elastic-dominant regime (tan δ < 1; G′ > G″) at approximately 74 °C. This shift marks the sol–gel transition, due to protein–protein interactions that drive the formation of a cohesive matrix [98]. Although several studies have reported an increase in the storage modulus (G′) upon addition of ChNCs into various protein and polysaccharide-derived matrices—such as soy protein isolate gels [99], starch composites [77], and PHB nanocomposites [96]—the addition of low ChNCs concentrations (1–2%) to the EWP-FFS did not lead to significant alterations in G′ or in the crossover temperature. However, at 5% ChNCs, the G′–G″ crossover occurred at lower temperatures, suggesting that ChNCs promote earlier structural network formation. This behavior may be attributed to their ability to act as physical crosslinking sites via hydrogen bonding, hydrophobic interactions, or even electrostatic interactions, thereby accelerating the gelation process and reinforcing the viscoelastic matrix.

### 3.6. Mechanical Properties

**Figure 5:**
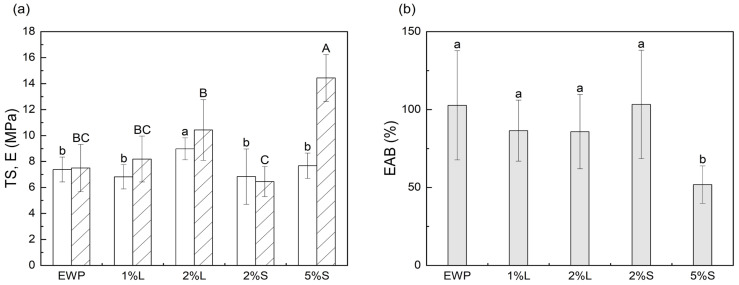
Effect on tensile strength (TS, white), elastic modulus (E, striped background) (**a**) and elongation at break (EAB) (**b**) of the reinforcement of egg white protein films with ChNCs in liquid (L) and solid (S) states at different concentrations. Data are presented as mean values (n = 15) with error bars indicating standard deviation. Significant differences (*p* < 0.05) among films are denoted by lowercase letters for TS and EAB, and uppercase letters for E.

Figure 5 presents the mean values of the mechanical properties of the analyzed replicates, obtained from films prepared in three batches, with no statistically significant differences detected among batches for the same ChNC concentration. The tensile strength (TS) of EWP films ranged from 6.83 to 8.98 MPa (Figure 5a). The incorporation of ChNCs at 2 wt.% via aqueous suspension significantly improved TS (from 7.38 to 8.98 MPa), likely due to improved nanoparticle dispersion and greater morphological homogeneity. Films produced with alternative incorporation methods or different ChNC concentrations showed no significant differences compared to neat EWP film. Regardless of the ChNC incorporation mode, their presence at 1 wt.% and 2 wt.% did not significantly affect the elastic modulus (E) or elongation at break (EAB) (Figure 5a,b). However, increasing the ChNC content to 5 wt.% led to statistically significant changes (*p* < 0.05), doubling E (from 7.8 ± 2.1 to 15.7 ± 5.3 MPa) and halving EAB (from 102.7 ± 35.0% to 51.9 ± 12.0%), yielding films that were stiffer and less flexible. These findings indicate that the incorporation of ChNCs markedly alters the mechanical behavior of EWP-based nanocomposite films. The observed changes can be attributed to both the reinforcing effect of rigid, crystalline ChNCs and their interaction with the protein network, likely through hydrogen bonding. Similar mechanical enhancements have been reported in other protein-based films containing ChNCs, such as gelatin [24,56], zein [58], and zein/potato starch nanocomposites [59]. Mechanical improvements have also been reported in EWP films reinforced with other nanoparticles, such as nanoclays, including higher tensile strength and enhanced strain hardening. These effects are associated with the limited mobility of polymer chains during stretching, induced by the intercalation of nanoparticles [65,68]. Similarly, films based on gelatin-EWP blends incorporating sepiolite exhibited a pronounced rise in E and TS, together with a notable decrease in EAB [69].

### 3.7. Optical Properties

Table 1 summarizes the CIELab color parameters and light transmittance values of the EWP films. ChNC incorporation had no significant influence on the L* and a* values or on the overall transmittance. However, a statistically significant rise in the b* value was detected with increasing ChNC content, becoming evident at concentration ≥2 wt.%. The observed increase in yellowness may be attributed to the intrinsic color of the chitin extracts, which is strongly influenced by both their biological origin and the extraction method employed [100]. These findings are in agreement with earlier studies on ChNC-reinforced films. For instance, in carboxymethyl cellulose films, ChNC incorporation up to 5 wt.% had no impact on L*, while b* values increased [80]. Similar trends were reported for corn starch films, where L* remained stable and b* increased with ChNC loading up to 5 wt.% [89]. The addition of ChNCs at levels of up to 4 wt.% did not significantly affect the CIELab* color coordinates of gelatin films [24]. No significant changes in transmittance were observed at concentrations ≤5 wt.%; however, other studies have reported increased opacity at higher ChNC contents. Etxabide et al. [24] and Qin et al. [89] noted a reduction in transparency with rising ChNC concentrations, and more recent findings suggest that opacity increases more markedly when concentrations exceed 10 wt.% [87].

### 3.8. Barrier Properties

**Figure 6:**
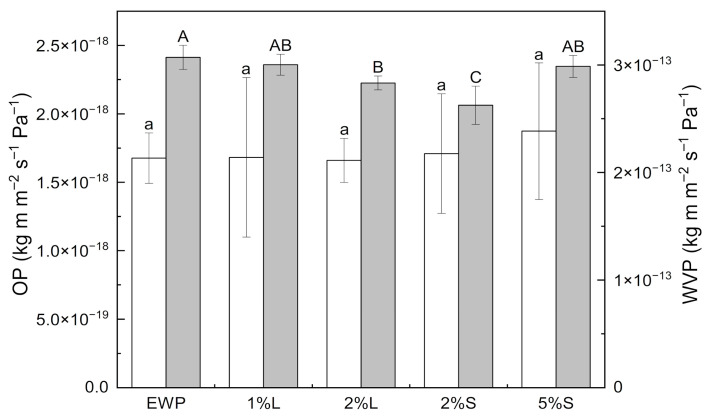
Effect on oxygen permeability (OP, white) and water vapor permeability (WVP, gray) of the reinforcement of egg white protein films with ChNCs in liquid (L) and solid (S) states at different concentrations. Data are presented as mean values (n = 15) with error bars indicating standard deviation. Significant differences (*p* < 0.05) among films are denoted by lowercase letters for WVP and uppercase letters for OP.

Figure 6 shows the mean values of the barrier properties of the analyzed replicates, with no statistically significant differences observed among batches for the same ChNC concentration. The permeabilities to oxygen and water vapor in the neat EWP films were 1.68 ± 0.18 × 10^−18^ kg m m^−2^ s^−1^ Pa^−1^ and 3.07 ± 0.11 × 10^−13^ kg m m^−2^ s^−1^ Pa^−1^, respectively. These values are consistent with previously reported data for EWP-based films [64,101,102].

Reinforcement with ChNCs did not significantly alter the OP across the studied concentration range (1–5 wt.%). Similar outcomes have been reported in PLA [103] and starch matrices at ChNC contents ≤5 wt.%. However, reductions in OP have been observed at higher loadings [84], or in more interactive polymer matrices, such as gelatin, where significant decreases occurred at ChNC levels below 0.5 wt.% [55], or in albumen-based films reinforced with montmorillonite clay [68]. When observed, such barrier improvements are typically attributed to the ability of nanofillers to occupy intermolecular voids and establish hydrogen bonds, thereby limiting gas permeation.

Regarding WVP, incorporation of 1 wt.% ChNCs (in aqueous suspension) did not induce significant changes. However, at 2 wt.%, WVP was reduced by 14.5% when ChNCs were incorporated as powder and by 7.8% when added as aqueous dispersion. These improvements are likely due to multiple factors: increased tortuosity of the diffusion pathway, enhanced biopolymer crystallinity, and the formation of a dense interpenetrating network through hydrogen bonding and physical entanglement between the nanocrystals and the protein matrix [56,77,80]. At 5 wt.% ChNCs, the WVP returned to values statistically equivalent to the neat EWP film, indicating a loss of the previously observed barrier enhancement. This behavior, also described for starch-based films [89], is likely due to nanoparticle aggregation and/or excess amino groups (–NH_2_), which are more hydrophilic than hydroxyl groups (–OH) and therefore increase water affinity [84]. Improvements in water barrier properties due to ChNC incorporation have been reported for a variety of polymer matrices, including gelatin [56], carrageenan [104], chitosan [88], starch [77,84,105], carboxymethyl cellulose [80], PLA [103,106,107], and PBS [108]. In EWP-based films, improved WVP has also been achieved using nanoclays, yielding reductions of up to 41% [69], although no such effect had been previously reported for ChNCs.

## 4. Conclusions

In this study, bio-nanocomposite films based on EWP and ChNCs were developed using both aqueous suspensions and powder forms. The incorporation of ChNCs produced materials with different mechanical properties: films with 2 wt.% ChNCs in suspension showed higher tensile strength while retaining flexibility, whereas those with 5 wt.% in powder form exhibited increased stiffness (Young’s modulus) at the expense of reduced elongation.

Structural analyses by FTIR and XRD confirmed the preservation of protein-specific bands and the enhancement of crystallinity, particularly in films with ChNCs in powder form, while SEM revealed better nanoparticle dispersion in those prepared with ChNCs in suspension. TGA results indicated a slight reduction in thermal stability at higher ChNC contents, likely due to nanoparticle aggregation. Regarding optical properties, reinforcement did not significantly affect transparency or lightness, although an increase in the yellowness (b* value) was observed. In terms of barrier performance, oxygen permeability remained unchanged, while water vapor permeability was reduced by up to 14.5% in the best performing formulation. These findings highlight the crucial role of the ChNCs incorporation method in determining the microstructure and overall performance of EWP-based films processed by compression molding. Nonetheless, some limitations persist, such as the limited improvement in gas barrier properties and the slight decrease in thermal stability.

Future work should address these shortcomings through complementary strategies, such as enzymatic crosslinking (e.g., with transglutaminase) or hydrophobic coatings to further improve water resistance and barrier performance. Moreover, extending the study to real storage conditions and evaluating biodegradability and performance in food packaging applications would strengthen the potential of EWP-based nanocomposite films as sustainable alternatives.

## Figures and Tables

**Table 1:** CIELab* color space and transmittance of egg white protein films with ChNCs in liquid (L) and solid (S) states at different concentrations.

Film Sample	L*	a*	b*	Transmittance (%)
EWP	96.72 ± 0.25 ^a^	−0.66 ± 0.18 ^a^	3.97 ± 0.26 ^b^	72.69 ± 3.12 ^a^
1% L	96.58 ± 0.37 ^a^	−0.64 ± 0.30 ^a^	4.06 ± 0.41 ^b^	72.14 ± 2.29 ^a^
2% L	96.53 ± 0.17 ^a^	−0.78 ± 0.09 ^a^	4.56 ± 0.24 ^a^	74.28 ± 3.22 ^a^
2% S	96.55 ± 0.29 ^a^	−0.57 ± 0.21 ^a^	4.53 ± 0.21 ^a^	72.30 ± 3.18 ^a^
5% S	96.60 ± 0.43 ^a^	−0.83 ± 0.14 ^a^	4.76 ± 0.28 ^a^	70.59 ± 2.74 ^a^

^a,b^ Different letters for the same parameter indicate significant differences between films (*p* < 0.05).

## Data Availability

The original data presented in the study are openly available in Zenodo at DOI: https://doi.org/10.5281/zenodo.17153286.

## Supplementary Materials

The following supporting information can be downloaded at: https://www.mdpi.com/article/10.3390/polym17182538/s1, Figure S1: Scanning electron microcopy (SEM) pictures of the surface of egg white protein films with ChNCs in liquid (L) and solid (S) states at different concentrations; Figure S2: Transmission electron microscopy (TEM) pictures of the cross-section of egg white protein films with ChNCs in solid state at 2% concentration.
