# Supplementary figures and images for: Novel Egg White Protein–Chitin Nanocrystal Biocomposite Films with Enhanced Functional Properties
Source: Polymers (Basel). 2025 Sep 19;17(18):2538. doi: 10.3390/polym17182538 (PMC12473369; doi:10.3390/polym17182538)

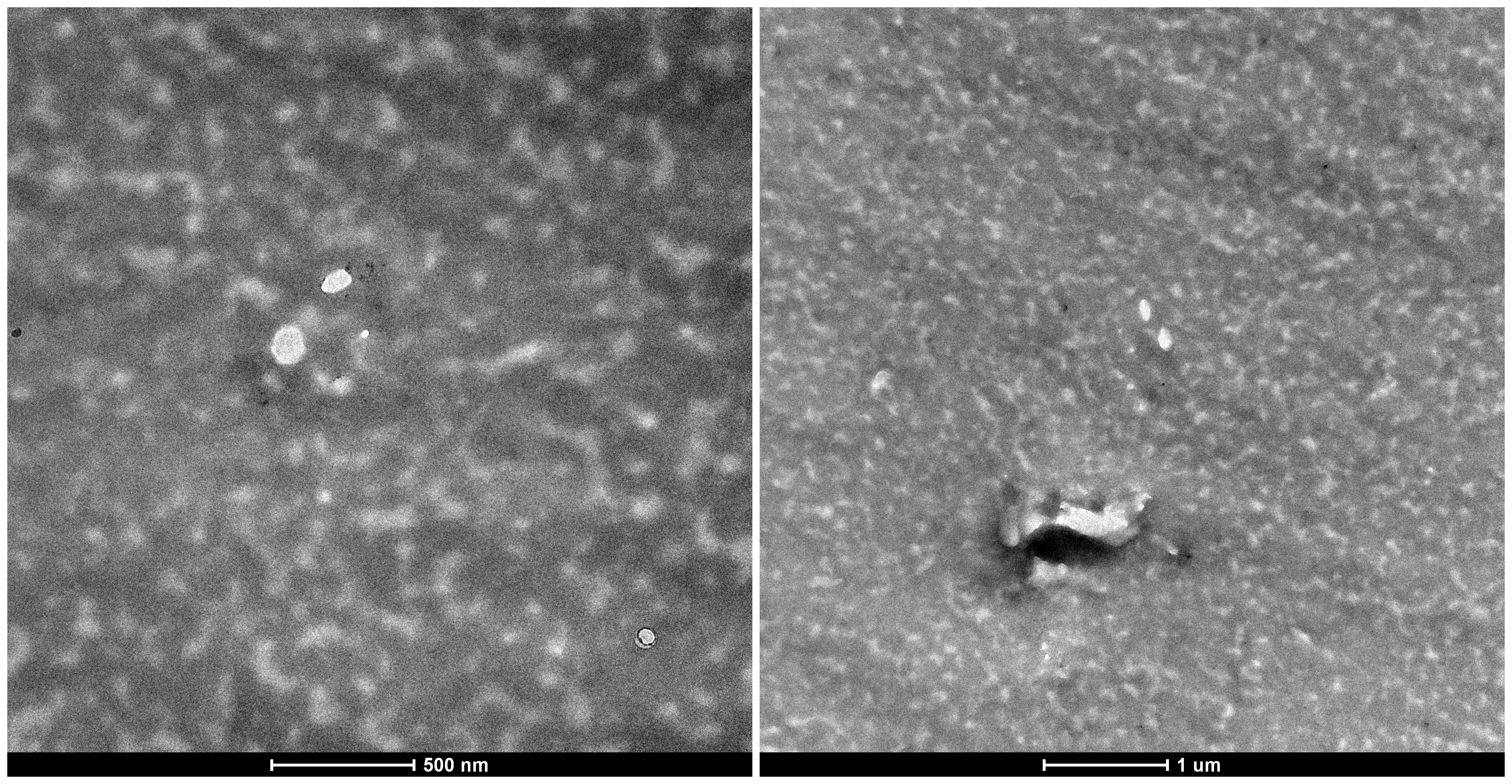

Supplement: Supplementary file 1 [file polymers-17-02538-s001.zip › FIGURE S2.tif]

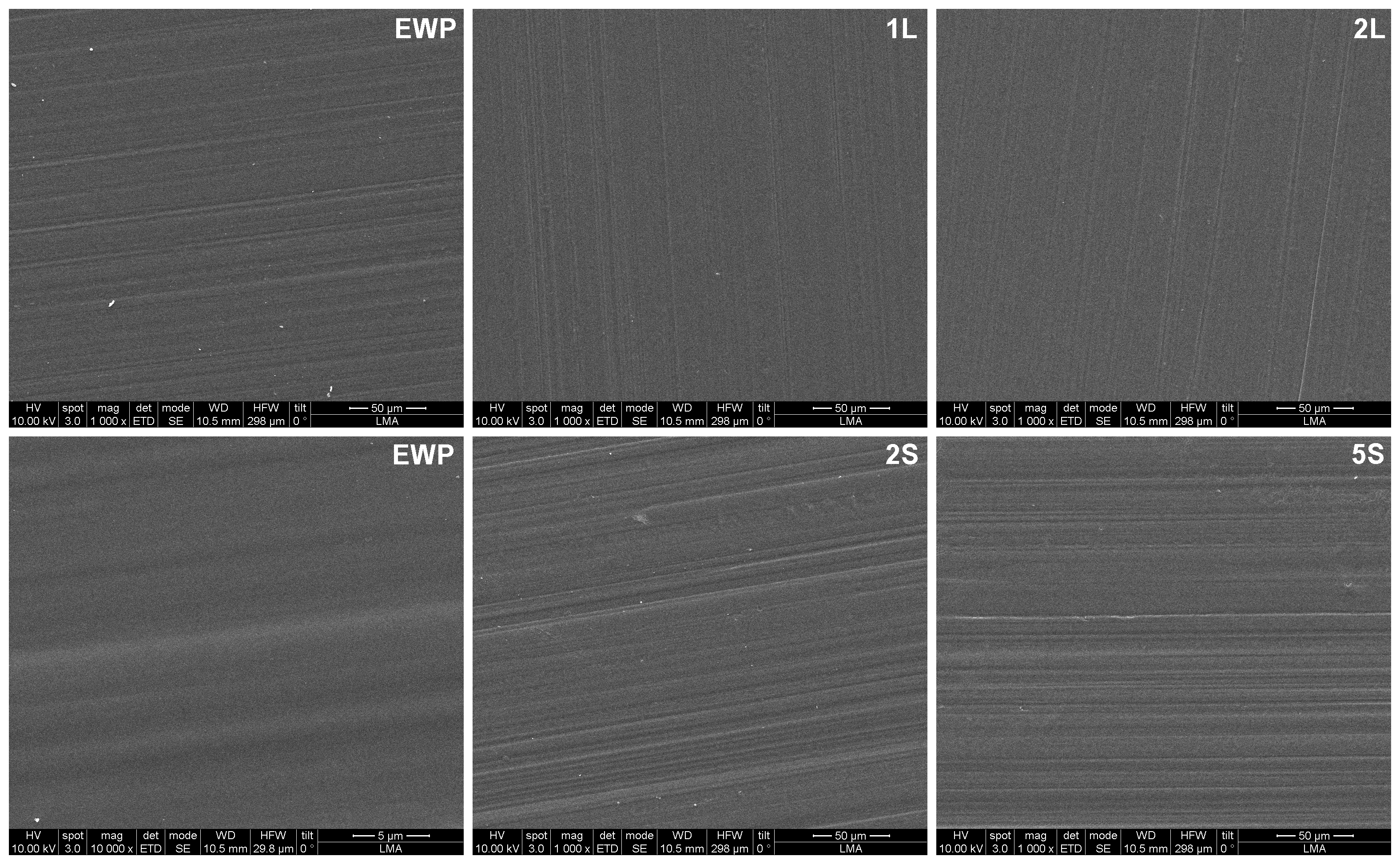

Supplement: Supplementary file 1 [file polymers-17-02538-s001.zip › FIGURE S1.tif]
